# Comparison of depression prevalence estimates in meta-analyses based on screening tools and rating scales versus diagnostic interviews: a meta-research review

**DOI:** 10.1186/s12916-019-1297-6

**Published:** 2019-03-21

**Authors:** Brooke Levis, Xin Wei Yan, Chen He, Ying Sun, Andrea Benedetti, Brett D. Thombs

**Affiliations:** 1 Lady Davis Institute for Medical Research, Jewish General Hospital, 4333 Cote Ste Catherine Road, Montreal, Quebec Canada; 2 Department of Epidemiology, Biostatistics and Occupational Health, McGill University, Montreal, Quebec Canada; 3 Respiratory Epidemiology and Clinical Research Unit, McGill University Health Centre, Montreal, Quebec Canada; 4 Department of Psychiatry, McGill University, Montreal, Quebec Canada; 5 Department of Medicine, McGill University, Montreal, Quebec Canada; 6 Department of Psychology, McGill University, Montreal, Quebec Canada; 7 Department of Educational and Counselling Psychology, McGill University, Montreal, Quebec Canada

**Keywords:** Depression, Prevalence, Meta-analysis, Classification methods, Transparency

## Abstract

**Background:**

Depression symptom questionnaires are commonly used to assess symptom severity and as screening tools to identify patients who may have depression. They are not designed to ascertain diagnostic status and, based on published sensitivity and specificity estimates, would theoretically be expected to overestimate prevalence. Meta-analyses sometimes estimate depression prevalence based on primary studies that used screening tools or rating scales rather than validated diagnostic interviews. Our objectives were to determine classification methods used in primary studies included in depression prevalence meta-analyses, if pooled prevalence differs by primary study classification methods as would be predicted, whether meta-analysis abstracts accurately describe primary study classification methods, and how meta-analyses describe prevalence estimates in abstracts.

**Methods:**

We searched PubMed (January 2008–December 2017) for meta-analyses that reported pooled depression prevalence in the abstract. For each meta-analysis, we included up to one pooled prevalence for each of three depression classification method categories: (1) diagnostic interviews only, (2) screening or rating tools, and (3) a combination of methods.

**Results:**

In 69 included meta-analyses (81 prevalence estimates), eight prevalence estimates (10%) were based on diagnostic interviews, 36 (44%) on screening or rating tools, and 37 (46%) on combinations. Prevalence was 31% based on screening or rating tools, 22% for combinations, and 17% for diagnostic interviews. Among 2094 primary studies in 81 pooled prevalence estimates, 277 (13%) used validated diagnostic interviews, 1604 (77%) used screening or rating tools, and 213 (10%) used other methods (e.g., unstructured interviews, medical records). Classification methods pooled were accurately described in meta-analysis abstracts for 17 of 81 (21%) prevalence estimates. In 73 meta-analyses based on screening or rating tools or on combined methods, 52 (71%) described the prevalence as being for “depression” or “depressive disorders.” Results were similar for meta-analyses in journals with impact factor ≥ 10.

**Conclusions:**

Most meta-analyses combined estimates from studies that used screening tools or rating scales instead of diagnostic interviews, did not disclose this in abstracts, and described the prevalence as being for “depression” or “depressive disorders ” even though disorders were not assessed. Users of meta-analyses of depression prevalence should be cautious when interpreting results because reported prevalence may exceed actual prevalence.

**Electronic supplementary material:**

The online version of this article (10.1186/s12916-019-1297-6) contains supplementary material, which is available to authorized users.

## Background

Validated diagnostic interviews are designed to be used in research to replicate diagnostic criteria and facilitate accurate classification of diagnostic status [1–8]. Prior to the 1980s, diagnostic classification of psychiatric disorders in research, including major depression, was done almost exclusively via unstructured clinician interviews [1–3]. The poor reliability of unstructured interviews, however, led to the development and validation of semi-structured and fully structured diagnostic interviews. Since then, many studies have demonstrated the improved performance of validated diagnostic interviews compared to unstructured interviews for classifying cases [2–5]. Today, it is expected that validated diagnostic interviews be used for major depression classification in research, including for the purpose of estimating prevalence [6, 7].

Administration of validated diagnostic interviews is time and resource intensive, however. Thus, instead of validated diagnostic interviews, researchers sometimes use self-report depression symptom questionnaires, or screening tools, and report the percentage of patients above standard screening cutoff thresholds as prevalence [8]. Depression symptom questionnaires have important uses. They are commonly used for the assessment of symptom severity, regardless of diagnostic status, and as screening tools to identify people who may have depression based on scores above cutoff thresholds. When used as screening tools, they apply score-based cutoff thresholds to classify patients as positive or negative screens. These thresholds are calibrated to maximize sensitivity and specificity for screening, but not for classification of disorder or, in aggregate, to estimate the prevalence of disorder based on diagnostic criteria.

Theoretically, based on sensitivity and specificity estimates, screening tools would be expected to exaggerate prevalence compared to rates based on diagnostic criteria [8], although the degree to which one would expect this to be the case would depend on the specific screening tool and cutoff used. Because the false positive rate of screening tools is disproportionately high in lower prevalence populations, such as primary health care, estimated prevalence based on screening tools would be expected to be exaggerated most when true prevalence is lowest [8]. Table 1 shows the percentage of patients who would theoretically score above standard cutoffs for screening for commonly used depression screening tools based on sensitivity and specificity estimates from meta-analyses for each screening tool and for true prevalence of 5%, 10%, and 15% [9–12]. No studies, however, to the best of our knowledge, have examined how often screening tools are used to estimate the prevalence of major depression or depressive disorders in published research and if this results in higher estimates of prevalence compared to research based on diagnostic interviews that replicate standard diagnostic criteria.

**Table 1:** Comparison of true depression prevalence and expected percentage of patients above a cutoff based on sensitivity and specificity from commonly used depression screening tools

True prevalence (%)	Sensitivity	Specificity	% above screening test cutoff	% above test cutoff—true prevalence	Ratio of % above test cutoff/true prevalence
Patient Health Questionnaire-9 ≥ 10 [9]					
5%	78%	87%	16%	11%	3.3
10%	78%	87%	20%	10%	2.0
15%	78%	87%	23%	8%	1.5
Hospital Anxiety and Depression Scale ≥ 8 [10]					
5%	82%	74%	29%	24%	5.8
10%	82%	74%	32%	22%	3.2
15%	82%	74%	34%	19%	2.3
Hospital Anxiety and Depression Scale ≥ 11 [10]					
5%	56%	92%	10%	5%	2.1
10%	56%	92%	13%	3%	1.3
15%	56%	92%	15%	0%	1.0
Edinburgh Postnatal Depression Scale ≥ 12 [11]					
5%	86%	87%	17%	12%	3.3
10%	86%	87%	20%	10%	2.0
15%	86%	87%	24%	9%	1.6
Geriatric Depression Scale-15 ≥ 5 [12]					
5%	89%	77%	26%	21%	5.3
10%	89%	77%	30%	20%	3.0
15%	89%	77%	33%	18%	2.2

Meta-analyses are cited more than any other study design, and evidence from meta-analyses is prioritized in clinical practice guidelines [13, 14]. If prevalence estimates were inflated in meta-analyses due to overestimation based on cutoffs designed for screening with self-report questionnaires, this would misinform evidence users, including healthcare decision-makers. There are numerous examples of recently published meta-analyses of depression prevalence that have relied primarily on depression screening tools, including meta-analyses in very high-impact journals [15–18]. It is not known, however, how common this practice is, whether reported prevalence is greater when screening tools are used, and whether meta-analysis authors clearly report the classification methods used in studies pooled to generate prevalence estimates.

The objective of the present study was to review published meta-analyses of depression prevalence to determine (1) whether diagnostic interviews, screening or rating tools, or a combination of methods were used to classify depression in primary studies synthesized in meta-analyses; (2) if pooled prevalence values differed when based on primary studies that used diagnostic interviews only, screening or rating tools only, or a combination of methods; (3) if classification methods used in pooled studies were accurately described in meta-analysis abstracts; and (4) how meta-analysis abstracts described the synthesized prevalence estimates (e.g., major depression, depression, depressive symptoms). For objectives 3 and 4, we focused on what was reported in abstracts because many users read only the abstracts of journal articles [19–22].

## Methods

### Data sources and searches

We searched PubMed to identify a sample of meta-analyses on depression prevalence published in a 10-year period (January 1, 2008, through December 5, 2017), using the following search terms:

*((((depression[Title/Abstract] OR depressive[Title/Abstract] OR depressed[Title/Abstract])) AND meta-analysis[Title/Abstract]) AND (prevalence[Title/Abstract] OR rate[Title/Abstract] OR rates[Title/Abstract])) AND (“2008”[Date - Publication]*: *“3000”[Date - Publication])*.

### Study selection

We included articles in any language that (1) indicated in the title or abstract that they conducted a meta-analysis to determine the prevalence of depression, a depressive disorder, or depressive symptoms; (2) reported at least one pooled depression prevalence value in the abstract; and (3) included, either in the full text or in the supplementary files, a list of all meta-analyzed primary studies along with the depression classification methods used in each study (e.g., diagnostic interview, screening or rating tool, medical records). Eligible meta-analyses had to have documented that a systematic review was conducted and pooled results from at least two primary studies. Meta-analyses that reported prevalence for participants known to have mental disorders and meta-analyses on the diagnostic test accuracy of depression classification tools were excluded.

Search results were uploaded into DistillerSR, which was used to store and track search results and to track results of the review process. We used the duplicate detection function in DistillerSR to identify and remove potential duplicate citations that occurred, for instance, if there were updates to a previously published meta-analysis. In those cases, we retained only the most recently published update.

Two investigators independently reviewed titles and abstracts for eligibility. If either reviewer deemed a study potentially eligible, full-text article review was done by two investigators independently. Disagreement between reviewers after the full-text review was resolved by consensus, including consultation with a third reviewer as necessary. Detailed inclusion/exclusion coding guides are provided in Additional file 1: Methods S1–S2.

### Data extraction

For each included meta-analysis, we recorded the author, year of publication, journal, journal impact factor for the year of publication, and participant group for which prevalence values were extracted.

### Objectives 1 and 2: depression classification methods used and prevalence estimates

For each included meta-analysis, we recorded whether the abstract presented pooled depression prevalence estimates based on three categories of classification methods: (1) diagnostic interviews only (validated diagnostic interview or unstructured interview), (2) depression screening or rating tools only, or (3) a combination of diagnostic interviews, screening or rating tools, or other methods (e.g., medical records, self-report). See Additional file 1: Methods S3 for the coding guide used to classify methods.

For each meta-analysis, we then extracted data for up to one prevalence estimate from each of the three classification method categories. We extracted data for the first prevalence reported in the abstract for each category, with the following exceptions: (1) if the abstract presented prevalence values for an overall sample and subgroups, we prioritized the overall sample; (2) if the abstract presented prevalence values for multiple periods of prevalence (e.g., current, past year), we prioritized the most recent period; and (3) if the abstract reported prevalence for multiple diagnostic classifications (e.g., major depression, any depressive disorder), we prioritized major depression.

For each prevalence value that we extracted, from the full text of the meta-analysis or any published supplementary material, we recorded the number of studies pooled, the pooled sample size, and details on the classification methods used in each included primary study. For primary studies that used diagnostic interviews, we recorded whether they used a validated diagnostic interview versus an unstructured diagnostic interview. If it was not possible to determine from material published with the meta-analysis whether an included primary study used a validated versus unstructured interview, we extracted this from the primary study.

For meta-analysis articles that reported pooled prevalence based only on screening or rating tools or based on combined methods in the abstract, a prevalence estimate based on diagnostic interviews may have been generated, but de-emphasized. Thus, in these articles, we searched the full texts for a prevalence value based on diagnostic interviews.

### Objective 3: reporting in abstracts of classification methods used in pooled primary studies

For each meta-analysis, for each extracted prevalence value, we recorded the abstract terminology, if any, used to describe the types of classification methods used in pooled studies (e.g., diagnostic interviews only, screening or rating tools only, combination of methods).

### Objective 4: terminology used in abstracts to describe pooled prevalence values

For each extracted prevalence value in each meta-analysis, from the study abstract, we recorded the terminology used to describe the prevalence value (e.g., major depression, depression, depressive disorder, depressive symptoms, percentage above a cutoff).

For all objectives, one investigator extracted data from abstracts and published reports, and a second investigator reviewed and validated the extracted data using the DistillerSR Quality Control function. Any disagreements were resolved by consensus, including consultation with a third reviewer as necessary.

### Data synthesis and analysis

Our analysis was descriptive and aimed to report what meta-analysis authors did and reported at the meta-analysis level, but not to estimate actual prevalence, which was beyond the scope of our study and would have required different methodology.

We described the number of prevalence values identified and extracted for each depression classification method category and details on the classification methods used in the meta-analyses. When pooled prevalence values included primary studies that used diagnostic interviews, we described whether the diagnostic interviews were validated versus unstructured interviews.

For each category of depression classification method, we described the mean (standard deviation) and median (minimum, maximum) of extracted prevalence estimates and generated forest plots. Since our purpose was to describe reporting of prevalence estimates and not to estimate a pooled prevalence that could be applied to a particular participant group or setting, each prevalence estimate was weighted equally.

For meta-analysis articles that reported pooled prevalence values for multiple depression classification method categories in the abstract, we compared prevalence across categories. Similarly, for articles that did not report prevalence based on diagnostic interviews in the abstract, but did in the full text, we compared prevalence estimates.

In sensitivity analyses, we assessed whether findings were similar among meta-analyses published in high-impact journals (impact factor ≥ 10).

## Results

### Article selection

**Fig. 1:**
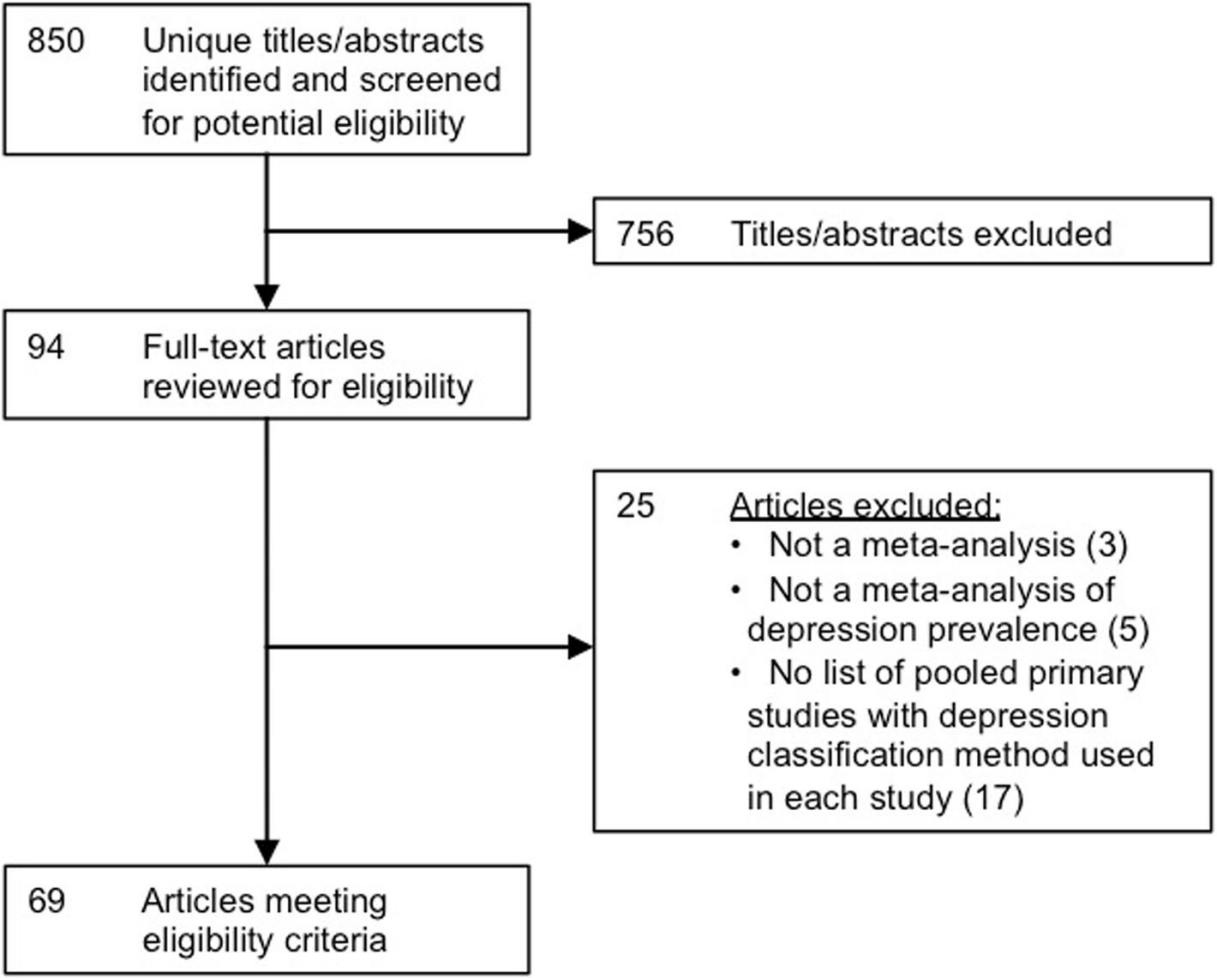
Flow diagram of the study selection process

The search retrieved 865 citations, of which 15 were duplicate citations. Of 850 unique citations, 756 were excluded after the title and abstract review and 25 after the full-text review. In total, 69 eligible articles were included (Fig. 1) from which 81 meta-analysis prevalence estimates reported in abstracts were included.

### Objective 1: classification methods used for depression prevalence estimates

As shown in Table 2, of 81 extracted prevalence estimates, eight (10%) were based on diagnostic interviews only, 36 (44%) on depression screening or rating tools only, and 37 (46%) on a combination of classification methods. In 12 meta-analysis articles that reported prevalence based on more than one classification category in the abstract, five estimated prevalence based on diagnostic interviews only and based on depression screening or rating tools only; seven estimated prevalence based on screening or rating tools only and based on a combination of classification methods.

**Table 2:** Summary of classification methods used in primary studies synthesized in meta-analyses for each depression classification method category (*N* meta-analyses = 69; *N* extracted prevalence values = 81)

	*N* (%) Validated diagnostic interviews	*N* (%) Unstructured diagnostic interviews	*N* (%) Screening or rating tools	*N* (%) Other methods	*N* total
Diagnostic interviews only (*N* = 8)	76 (72%)	29 (28%)	–	–	105 (100%)
Screening or rating tools only (*N* = 36)	–	–	759 (100%)	–	759 (100%)
Combination of classification methods (*N* = 37)	201 (16%)	57 (5%)	845 (69%)	127 (10%)	1230 (100%)
Total	277 (13%)	86 (4%)	1604 (77%)	127 (6%)	2094 (100%)

Five meta-analysis abstracts reported depression prevalence based on diagnostic interviews plus based on screening or rating tools only, and seven meta-analysis abstracts reported depression prevalence based on a combination of classification methods plus based on screening or rating tools only

Of the eight meta-analyses based on diagnostic interviews, four (50%) only included studies that used validated diagnostic interviews, whereas four also included studies that used unstructured clinician interviews. Overall, 76 of 105 (72%) primary studies in the eight meta-analyses used validated diagnostic interviews to classify depression. In 37 meta-analyses based on a combination of classification methods, 201 of 1230 included primary studies (16%) used validated diagnostic interviews to classify depression. Overall, among 2094 primary studies included in the 81 pooled prevalence estimates, 277 (13%) used validated diagnostic interviews, 1604 (77%) used screening or rating tools, and 213 (10%) used other methods (e.g., unstructured interviews, medical records, self-report; Table 2).

See Additional file 1: Tables S1a–c for characteristics of meta-analyses based on each classification method.

### Objective 2: pooled prevalence estimates by depression classification method category

Mean pooled depression prevalence was 17% (median 15%) for meta-analyses based on diagnostic interviews, 31% (median 30%) based on screening or rating tools, and 22% (median 23%) based on a combination of methods (Table 3). See Additional file 1: Figures S1a–c for forest plots of pooled prevalence estimates from meta-analyses based on each classification method.

**Table 3:** Prevalence estimates in meta-analyses for each depression classification method category (*N* meta-analyses = 69, *N* extracted prevalence values = 81)

		*N* included primary studies	*N* pooled participants	Pooled prevalence (%)
Diagnostic interviews only (*N* = 8)	Median (range)	5 (2 to 49)	3093 (299 to 11,286)	15 (7 to 31)
	Mean (SD)	13 (17)	4043 (3902)	17 (9)
Screening or rating tools only (*N* = 36)	Median (range)	17 (2 to 81)	7236 (659 to 442,482)	30 (9 to 62)
	Mean (SD)	21 (17)	27,487 (74,504)	31 (13)
Combination of classification methods (*N* = 37)	Median (range)	21 (3 to 183)	19,468 (197 to 495,229)	23 (1 to 48)
	Mean (SD)	33 (41)	47,361 (89,237)	22 (12)

Five meta-analysis abstracts reported depression prevalence based on diagnostic interviews plus based on screening or rating tools only, and seven meta-analysis abstracts reported depression prevalence based on a combination of classification methods plus based on screening or rating tools only

*Abbreviations*: *SD* standard deviation

For the five meta-analyses that reported prevalence in the abstract for both diagnostic interviews plus screening or rating tools and the seven that reported for both a combination of methods plus screening or rating tools, prevalence was always greater based on screening or rating tools (mean difference = 10 percentage points). See Additional file 1: Figures S2a-b.

Eight meta-analyses did not report a prevalence value based on diagnostic interviews in the abstract but provided one in the article text. In all eight, the prevalence reported in the abstract from screening or rating tools or from a combination of methods was greater than the prevalence estimate based on diagnostic interviews that was not reported in the abstract (mean difference = 7 percentage points).

### Objective 3: reporting in abstracts of classification method categories in pooled primary studies

**Fig. 2:**
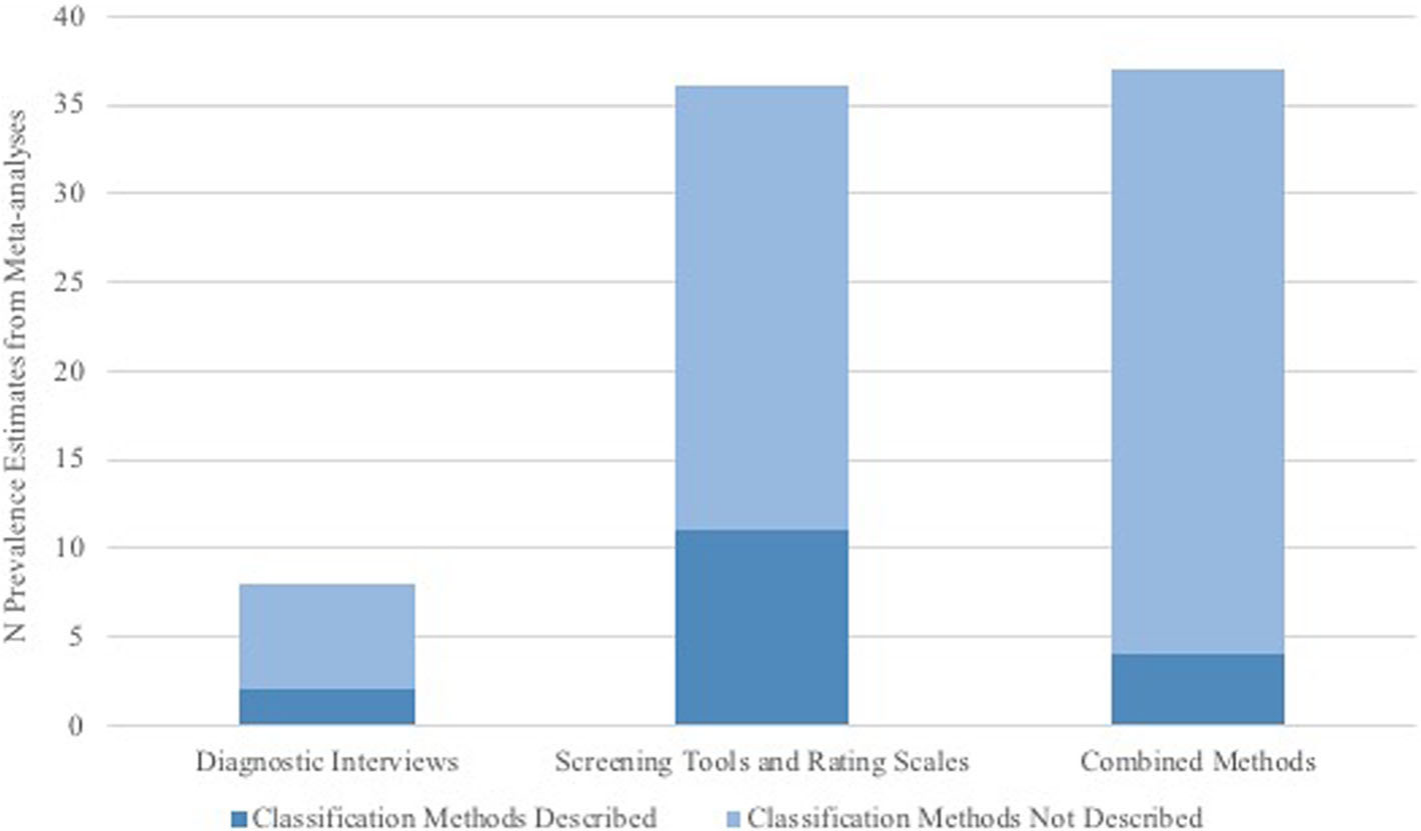
Number of meta-analyses per classification category and whether abstracts described the depression classification methods pooled

Only two of eight (25%) abstracts with prevalence estimates based on diagnostic interviews noted that the meta-analysis pooled only studies that used diagnostic interviews; the other six (75%) did not describe classification methods. For prevalence values based on screening or rating tools only, 11 of 36 abstracts (31%) indicated that the meta-analysis combined studies that used screening or rating tools, while two (6%) used the terms “a structured tool” or “a validated instrument,” and 23 (64%) did not describe classification methods. For prevalence values based on a combination of classification methods, only four of 37 abstracts (11%) reported that the meta-analysis pooled studies that used a combination of classification methods, while six (16%) used terms such as “interview,” “diagnostic codes,” or “clinician diagnosis,” and 27 (73%) did not describe classification methods. In total, only 17 of 81 (21%) prevalence estimates included an accurate description of the classification methods used in pooled primary studies (Fig. 2).

### Objective 4: terminology used in abstracts to describe pooled prevalence values

For prevalence values based on diagnostic interviews only, all eight abstracts (100%) referred to the prevalence as being for “depression” or “depressive disorders.” For prevalence values based on screening or rating tools only, 21 of 36 abstracts (58%) referred to the prevalence as being for “depression” or “depressive disorders,” whereas 11 (31%) used the term “depressive symptoms,” one (3%) used the term “depression or depressive symptoms,” one (3%) used the term “clinically significant depressive symptoms,” one (3%) used the term “clinically significant levels of depression,” and one (3%) used the term “probable depression.” For prevalence values based on a combination of classification methods, 31 of 37 abstracts (84%) referred to the prevalence as being for “depression” or “depressive disorders,” four (11%) used the term “depression or depressive symptoms,” and two (5%) used the term “depressive symptoms.” Overall, among 73 prevalence estimates not based exclusively on diagnostic interviews, 52 (71%) nonetheless described the estimate as the prevalence of “depression” or “depressive disorders.”

### Sensitivity analysis of meta-analyses published in journals with impact factor ≥ 10

Seven meta-analyses were published in journals with impact factors ≥ 10 (Additional file 1: Table S1d). These articles were published in journals with impact factors from 18 to 44 for the years of publication. All seven articles reported only pooled prevalence based on a combination of classification methods. Of 365 primary studies included in the seven meta-analyses, 39 (11%) used a validated diagnostic interview to classify depression. Mean pooled prevalence was 20% (median 19%).

Two of the seven abstracts (29%) reported that the meta-analysis combined studies that classified depression using a combination of classification methods, while one (14%) used the term “psychiatric interviews” and four (57%) did not describe classification methods. Five of the seven abstracts (71%) referred to the prevalence value as being for “depression,” and two (29%) used the term “depression or depressive symptoms.”

## Discussion

We reviewed 69 published meta-analyses on depression prevalence, including 81 separate prevalence estimates. There were four main findings. First, only 10% of pooled prevalence estimates were based exclusively on primary studies that used diagnostic interviews to classify depression, and only half of these were restricted to validated diagnostic interviews. Among 2094 primary studies included in 81 prevalence estimates, only 13% used validated diagnostic interviews, and 77% used screening or rating tools (10% used other methods). Second, meta-analysis authors rarely disclosed this. Classification methods from pooled primary studies were described accurately in abstracts for only 21% of prevalence estimates. Third, 71% of meta-analyses that pooled results from screening or rating tools or from a combination of methods described the prevalence as being for “depression” or “depressive disorders” even though disorders were not assessed. Fourth, prevalence estimates based on depression screening or rating tools were on average 14% greater than estimates based on diagnostic interviews. Within meta-analyses, when prevalence was estimated with more than one classification method, prevalence based on screening or rating tools was on average 10% greater than other methods.

Depression accounts for more years of “healthy” life lost than any other medical condition [19–22]. Improving depression identification and management is an important challenge, and improving depression care is a global priority [23–27]. The etiology of depression is multifactorial and associated with many different risk factors, including poor physical health, job strain, trauma and loss, social economic factors, genetic factors, and others [28, 29]. Understanding differences in the prevalence of depression in different populations is important for making decisions about how best to address it. Estimating prevalence with inappropriate methods, however, misinforms evidence users, including health care decision-makers. It could also lead to misdiagnosis and treatment of non-depressed patients by clinicians who are led to believe that screening tools are diagnostic and can form the basis of treatment decisions [30].

When published in high-impact journals, misleadingly high prevalence estimates based on inappropriate classification methods can be highly influential and may distort efforts to address an important problem. As an example, a meta-analysis published in 2016 in *JAMA* [18] reported an overall pooled prevalence of what was labeled “depression or depressive symptoms” among medical students of 27%. This estimate, however, was based on depression symptom questionnaires in 182 of 183 included studies. The only included study that used a validated diagnostic interview [31] reported a prevalence of major depression of 9%, which is not substantively different than the 11% among 18 to 25-year-olds and 7% among 26 to 49-year-olds in the US general population [32]. Despite this, results from the meta-analysis were widely disseminated, and the meta-analysis was listed by Altmetric as among the top 100 “most-discussed” journal articles out of the 2.2 million research outputs tracked by Altmetric in 2017 [33]. Mental health and well-being are important concerns for medical trainees at all levels. Supporting trainees to cope with stress and effectively address mental health problems are important priorities [34]. The use of research methods that dramatically over-identify depression cases, however, makes it difficult to understand where needs are greatest, identify factors associated with the onset of mental health problems, and find effective solutions.

Some authors of meta-analyses label percentages of patients above cutoffs on screening tools or identified by other non-diagnostic methods as the prevalence of “depressive symptoms” or similar terms rather than “depression” or “depressive disorders.” This, however, does little to mitigate the problem. In these cases, results from different screening tools and cutoffs are often synthesized. There is no way to link such a pooled prevalence to any single method that could be reproduced in a specific clinical setting, since percentages above cutoffs vary dramatically depending on the screening tool and cutoff used. Furthermore, even if studies that all use the same screening tool are pooled in a meta-analysis, there is no evidence that classification cutoffs from screening questionnaires reflect a meaningful divide between impairment and non-impairment [8].

Given the importance of accurately estimating depression prevalence and the high level of resources needed to administer validated diagnostic interviews, less resource-intensive alternatives are desirable. We recently examined several options and found that researchers can obtain reasonably precise prevalence estimates by using a two-stage approach [8]. In this approach, first, all study participants are administered a screening tool. Then, all participants with positive screens, but only a random sample of those with negative screens, are evaluated with a diagnostic interview. In one example, in a sample of 1000 study participants and a true prevalence of 10%, interviewing only 10% of those with negative screening results resulted in a total of 28% of participants needing to be interviewed and a relatively small increase in the width of the 95% confidence interval from 3.7% if all 1000 received diagnostic interviews to 7.0% with 276 receiving interviews [8].

Another approach, prevalence matching, would involve calibrating cutoffs on depression symptom questionnaires to estimate case prevalence in a population rather than to maximize sensitivity and specificity for screening. This could be done by administering a screening tool and a validated diagnostic interview to all patients in a study and setting a cutoff score that results in the percentage above the cutoff matching as closely as possible the number of patients with depression, based on the validated diagnostic interview [8, 35]. We do not know, however, of any examples where this has been done.

The present study is the first to demonstrate that the vast majority of meta-analyses of depression prevalence are based on primary studies that use inappropriate depression classification methods known to inflate prevalence, that this information is not accurately described in meta-analysis abstracts, that prevalence values are most commonly described as “depression” or “depressive disorders” even when diagnostic interviews are not used, and that this distorts reported prevalence estimates substantially.

One limitation of our study was the extensive heterogeneity across meta-analyses. The purpose of our study was not to determine the true prevalence of depression in any particular participant group or setting, but to describe methods of synthesis and reporting. Beyond descriptive analyses, we did not attempt to conduct a meta-analysis of the magnitude by which depression screening or rating tools exaggerated depression prevalence due to the broad heterogeneity in the included meta-analyses, including participant populations, the range of different depression screening tools and cutoffs included within and across meta-analyses, and the different depressive disorders that were assessed when diagnostic interviews were used (e.g., major depression, any depressive disorder). Nonetheless, based on descriptive analyses, it appears that, consistent with what has been shown previously on a theoretical level [8], screening or rating tools generate prevalence estimates substantially greater than those obtained from diagnostic interviews. Future work should quantify the extent of prevalence inflation based on specific depression screening or rating tools by comparing prevalence based on a specific screening tool and cutoff in comparison to a specific diagnostic interview and disorder.

A second possible limitation is that we did not examine the differential performance of different types of validated diagnostic interviews as this was beyond the scope of the study. Indeed, there are differences in the performance of different validated diagnostic interviews, as we demonstrated in a recent meta-analysis [36], and different types of diagnostic interviews may be differentially reliable [37, 38]. Furthermore, these interviews may not be used in the way that they are intended or by the types of interviewers for who they are designed, which could also influence their performance.

## Conclusion

In summary, most existing meta-analyses of depression prevalence are based primarily on studies that used methods other than validated diagnostic interviews to classify depression, do not disclose in the abstract the classification methods used in pooled studies, and inaccurately refer to prevalence values as reflecting “depression” or “depressive disorders.” Researchers and policy makers who use meta-analyses of depression prevalence should refer to the full texts of meta-analyses to determine what methods were used and whether the abstract may have reported an inflated estimate.

## Additional file

Additional file 1: **Methods S1.** Title and abstract eligibility coding guide. **Methods S2.** Full-text eligibility coding guide. **Methods S3.** Categorization of depression classification methods. **Table S1.** a: Characteristics of included meta-analyses based on diagnostic interviews only. **Table S1.** b: Characteristics of included meta-analyses based on screening tools and rating scales only. **Table S1.** c: Characteristics of included meta-analyses based on a combination of classification methods (validated diagnostic interview, unstructured diagnostic interview, screening tool or rating scale, other—e.g., medical records). **Table S1.** d: Characteristics of included meta-analyses published in journals with impact factor ≥ 10 (all were based on a combination of classification methods (validated diagnostic interview, unstructured diagnostic interview, screening tool or rating scale, other—e.g., medical records)). **Figure S1.** a: Forest plot of pooled prevalence estimates from meta-analyses based on diagnostic interviews only. **Figure S1.** b: Forest plot of pooled prevalence estimates from meta-analyses based on screening tools and rating scales only. **Figure S1.** c: Forest plot of pooled prevalence estimates from meta-analyses based on a combination of classification methods (validated diagnostic interview, unstructured diagnostic interview, screening tool or rating scale, other—e.g., medical records). **Figure S2.** a: Forest plots of pooled prevalence estimates from studies with meta-analyses based on screening tools and rating scales only and meta-analyses based on diagnostic interviews only. **Figure S2.** b: Forest plots of pooled prevalence estimates from studies with meta-analyses based on screening tools and rating scales only and meta-analyses based on a combination of classification methods (validated diagnostic interview, unstructured diagnostic interview, screening tool or rating scale, other—e.g., medical records). (DOCX 1126 kb)

## Abbreviation

PHQ-9: Patient Health Questionnaire-9
