# Supplementary material for: Comparison of depression prevalence estimates in meta-analyses based on screening tools and rating scales versus diagnostic interviews: a meta-research review
Source: BMC Med. 2019 Mar 21;17:65. doi: 10.1186/s12916-019-1297-6 (PMC6427845; doi:10.1186/s12916-019-1297-6)
Supplement: Supplementary file 1 — Methods S1. Title and abstract eligibility coding guide. Methods S2. Full-text eligibility coding guide. Methods S3. Categorization of depression classification methods. Table S1. a: Characteristics of included meta-analyses based on diagnostic interviews only. Table S1. b: Characteristics of included meta-analyses based on screening tools and rating scales only. Table S1. c: Characteristics of included meta-analyses based on a combination of classification methods (validated diagnostic interview, unstructured diagnostic interview, screening tool or rating scale, other—e.g., medical records). Table S1. d: Characteristics of included meta-analyses published in journals with impact factor ≥ 10 (all were based on a combination of classification methods (validated diagnostic interview, unstructured diagnostic interview, screening tool or rating scale, other—e.g., medical records)). Figure S1. a: Forest plot of pooled prevalence estimates from meta-analyses based on diagnostic interviews only. Figure S1. b: Forest plot of pooled prevalence estimates from meta-analyses based on screening tools and rating scales only. Figure S1. c: Forest plot of pooled prevalence estimates from meta-analyses based on a combination of classification methods (validated diagnostic interview, unstructured diagnostic interview, screening tool or rating scale, other—e.g., medical records). Figure S2. a: Forest plots of pooled prevalence estimates from studies with meta-analyses based on screening tools and rating scales only and meta-analyses based on diagnostic interviews only. Figure S2. b: Forest plots of pooled prevalence estimates from studies with meta-analyses based on screening tools and rating scales only and meta-analyses based on a combination of classification methods (validated diagnostic interview, unstructured diagnostic interview, screening tool or rating scale, other—e.g., medical records). (DOCX 1126 kb) [file 12916_2019_1297_MOESM1_ESM.docx]

**Additional file 1**

**Methods 1. Title/Abstract Eligibility Coding Guide**

Question:

Is this potentially an eligible meta-analysis that states in the abstract that it is reporting prevalence of depression, a depressive disorder, or depressive symptoms?

Possible responses:

**No: Not a meta-analysis.** Exclude if it is clear from the title and abstract that the article is not a meta-analysis. Note that to count as a meta-analysis, a study must have (1) included a documented systematic review for the identification of eligible primary studies, and (2) pooled results from at least 2 primary studies.

**No: Not a meta-analysis of depression prevalence.** Exclude if it is clear from the title and abstract that the article does not report that it pooled estimates of prevalence of depression, a depressive disorder, or depressive symptoms.

**Note:** Meta-analyses that report prevalence of depression, a depressive disorder, or depressive symptoms for participants in studies of interventions or among patients with other mental disorders should be excluded. Meta-analyses on the diagnostic test accuracy of depression classification methods should also be excluded.

**No: No estimate of pooled prevalence reported in the abstract.** Exclude if the abstract does not include a numerical value of pooled prevalence of depression, a depressive disorder, or depressive symptoms.

**Yes: Potentially eligible meta-analysis.** Study eligible to be included in full-text review.

**Methods 2. Full-text Eligibility Coding Guide**

Question:

Is this an eligible meta-analysis that states in the abstract that it is reporting prevalence of depression, a depressive disorder, or depressive symptoms?

Possible responses:

**No: Not a meta-analysis.** Exclude if the article is not a meta-analysis. Note that to count as a meta-analysis, a study must have (1) included a documented systematic review for the identification of eligible primary studies, and (2) pooled results from at least 2 primary studies.

**No: Not a meta-analysis of depression prevalence.** Exclude if the article did not pool estimates of prevalence of depression, a depressive disorder, or depressive symptoms.

**Note:** Meta-analyses that report prevalence of depression, a depressive disorder, or depressive symptoms for participants in studies of interventions or among patients with other mental disorders should be excluded. Meta-analyses on the diagnostic test accuracy of depression classification methods should also be excluded.

**No: No list of pooled primary studies with depression classification method used in each study.** Exclude if the article does not provide a list of all meta-analysed primary studies along with the method used to classify case status (e.g., name of questionnaire [with or without cutoff threshold used], name of diagnostic interview).
**Note:** The list might appear in the supplementary files. If the article mentions supplementary materials, find them through PubMed, then upload to Distiller (refID_author_supplemental1) and use to help determine eligibility.

**Yes: Eligible meta-analysis.** Study eligible to be included in our review.

**Methods 3. Categorization of Depression Classification Methods**

Screening Tools and Rating Scales

**ADS-K** (General Depression Scale Short Form - in German)

**AKUADS** (Aga Khan University Anxiety and Depression Scale)

**APS** (Adolescent Psychopathology Scale) **BASDEC** (Brief Assessment Schedule Depression Cards)

**BDI** or **BDI II** (Beck Depression Inventory)

**Birleson Depression Self-Rating Scale**

**BMS** (Brief Mood Scale)

**BSI** (Brief Symptom Inventory)

**CDS** (Cardiac Depression Scale)

**CDI** (Children’s Depression Inventory) **CES-D** (Center for Epidemiologic Studies Depression Scale)

**CGI** (Clinical Global Impression Scale)

**CIDI-SF** (Composite International Diagnostic Interview – Short Form)

**CPRS** (Comprehensive Psychopathological Rating Scale)

**CRSD** (Carroll Rating Scale for Depression)

**DAWBA** (Development and Well-Being Assessment)

**DASS-21** and **DASS-42** (Depression Anxiety Stress Scale)

**DI** (Depression Inventory) **DSP** (Derogatis Stress Profile)

**DSRSC** (Depression Self-Rating Scale for Children)

**DSSI** (Delusions-Symptoms-States Inventory)

**DT** (Distress Thermometer)

**EST-Q (**Emotional State Questionnaire)

**EURO-D**

**GHQ** (General Health Questionnaire)

**GDS** (Geriatric Depression Scale)

**GMDS** (Gotland Male Depression Scale) **HADS** or **HADS-A** or **HADS-D** (Hospital Anxiety and Depression Scale)

**HAM-D / HDRS** (Hamilton Depression Rating Scale)

**HRSRS** (Health-Related Self-Reported Scale)

**(H)SCL-25** (Hopkins Symptom Check List)

**IDCL** (International Diagnostic Checklist for ICD-10)

**IDD** (Inventory to Diagnose Depression) **IES** (Impact of Events Scale)

**IWQOL-Lite** (Quality of Life-lite Questionnaire)

**K-10** (Kessler Psychological Distress Scale)

**KADS** (Kutcher Adolescent Depression Scale)

**MADRS** (Montgomery Asberg Depression Rating Scale – Depression subscale of the Comprehensive Psychopathological Rating Scale (**CPRS**))

**MDI** (Major Depression Inventory)

**MEQ** (Mood Evaluation Questionnaire)

**MHI-5** (Five-question Mental Health Inventory)

**MHI-38** (38-question Mental Health Inventory)

**MMPI-D** (Minnesota Multiphasic Personality Inventory-Depression Scale)

**MOOD SR-LM** (Mood Spectrum Self Report – Last Month)

**PAIS** (Psychosocial Adjustment to Illness Scale)

**PANAS** (Positive And Negative Affect Schedule

**PHQ-9** or **PHQ-8** or **PHQ-2** (Patient Health Questionnaire)

**POMS** (Profile of Mood States)

**PRIME-MD** (Primary Care Evaluation of Mental Disorders)

**PROMIS-T** (Patient-Reported Outcomes Measurement Information System)

**QIDS** (Quick Inventory of Depressive Symptomatology)

**RDRS** (Raskin Depression Rating Scale)

**Rutter Children Behaviour Questionnaire**

**SAI** (State Anxiety Inventory)

**SCL-90** (Symptoms Checklist 90)

**SF-36** (Short Form-36 – Mood subscale)

**SNASA** (Salford Needs Assessment Schedule for Adolescents)

**SRQ** (Self-Reporting Questionnaire) **SRQ-20** (Self-Reporting Questionnaire – 20 item) **SSI** (Symptoms of Stress Inventory)

**TDI** (Thai Depression Inventory)

**WDI** (Wakefield Depression Inventory) **WHO Major Depression Inventory**

**Whooley questions**

**ZSDS** (Zung Self-rating Depression Scale)

Validated semi-structured or fully structured diagnostic interviews

**ADIS-IV** (Anxiety Disorders Interview Schedule-IV)

**AUDADIS-IV** (The Alcohol Use Disorder and Associated Disabilities Interview Schedule-IV)

**CAPA** (Child and Adolescent Psychiatric Assessment) **CAS** (Child Assessment Schedule)

**C-DIS** (Computerized Diagnostic Interview Schedule)

**CIDI** (Composite International Diagnostic Interview)

**CIDIS** (Composite International Diagnostic Interview Simplified) **CIS** (Clinical Interview Schedule)

**CIS-R** (Clinical Interview Schedule - Revised)

**DICA** (Diagnostic Interview for Children and Adolescents)

**DIGS** (Diagnostic Interview for Genetic Studies)

**DIS** (Diagnostic Interview Schedule)

**DISC** (Diagnostic Interview Schedule for Children)

**DISH** (Diagnostic Interview and Structured Hamilton)

**DQPD** (Diagnostic Questionnaire for Depressive Patients)

**GMS** (Geriatric Mental State – Community Version or **AGECAT** Version)

**K-SADS** (SADS for school-aged children)

**Kinder-DIPS** (Diagnostisches Interview bei psychischen Störungen im Kindes)

**MILP** (Monash Interview for Liaison Psychiatry)

**MINI** (Mini International Neuropsychiatric Interview)

**PADDI** (Practical Adolescent Dual Diagnostic Interview) **PAS** (Psychiatric Assessment Schedule)

**PSE** (Present State Examination)

**RCIS** (Revised Clinical Interview Schedule)

**SADS** (Schedule for Affective Disorders and Schizophrenia)

**SCAN** (Schedule for Clinical Assessment in Neuropsychiatry)

**SCID** (Structured Clinical Interview for DSM)

**SPI** (Standardized Psychiatric Interview)

**Table S1a. Characteristics of Included Meta-analyses based on Diagnostic Interviews Only**

| **First author, year** | **Journal** | **Impact factor for year of publication** | **Participant group** | **Classification method terminology in abstract** | **Terminology for prevalence value in abstract** | **N primary studies included** | **N participants** | **Pooled prevalence (%)** | **N (%) included studies with validated diagnostic interview** |
| --- | --- | --- | --- | --- | --- | --- | --- | --- | --- |
| **Barcelos-Ferreira, 2010 [1]**^a^ | International Psychogeriatrics | 2.5 | Community-dwelling elderly Brazilians | NR | Major depression | 4 | 1,163 | 7 | 2 (50) |
| **Bronsard, 2016 [2]** | Medicine | 1.8 | Children and adolescents in the child welfare system | NR | Depressive disorders | 5 | 1,339 | 11 | 5 (100) |
| **Krebber, 2014 [3]**^a^ | Psycho-Oncology | 4.0 | Adult cancer patients during or after treatment | (Semi) structured diagnostic interviews | Depression | 49 | 8,747 | 13 | 31 (63) |
| **Ojagbemi, 2017 [4]** | Behavioural Neurology | 2.1 | Poststroke patients in Sub-Saharan Africa | NR | Depression | 3 | 299 | 31 | 3 (100) |
| **Scott, 2017 [5]** | Epilepsia | 5.1 | People with epilepsy | NR | Depressive disorders | 27 | 3,225 | 23 | 21 (78) |
| **Silva, 2014 [6]**^a^ | Revista Brasileira de Psiquiatria | 1.8 | Brazilian adults | NR | Major depressive disorders | 5 | 11,286 | 8 | 5 (100) |
| **Volkert, 2013 [7]**^a^ | Ageing Research Reviews | 7.6 | Older people in Western countries | NR | Lifetime major depression | 2 | 3,324 | 17 | 2 (100) |
| **Zhang, 2017 [8]^a^** | BMC Psychiatry | 2.4 | Adult systemic lupus erythematosus patients | Clinical interviews | Major depression | 10 | 2,960 | 24 | 7 (70) |

**Abbreviations**: NR: Not reported.

^a^ Also included in Additional Table 1b (Meta-analyses based on depression screening tools and rating scales only).

**Table S1b. Characteristics of Included Meta-analyses based on Screening Tools and Rating Scales Only**

| **First author, year** | **Journal** | **Impact factor for year of publication** | **Participant group** | **Classification method terminology in abstract** | **Terminology for prevalence value in abstract** | **N primary studies included** | **N participants** | **Pooled prevalence (%)** |
| --- | --- | --- | --- | --- | --- | --- | --- | --- |
| **Abajobir, 2016 [9]** | Journal of Affective Disorders | 3.4 | Women with unintended pregnancy | NR | Depression | 10 | 17,556 | 21 |
| **Bao, 2017 [10]** | Neuroscience and Biobehavioral Reviews | 8.0 | Community-dwelling older adults | NR | Depressive symptoms | 24 | 84,520 | 18 |
| **Barcelos-Ferreira, 2010 [1]^a^** | International Psychogeriatrics | 2.5 | Community-dwelling elderly Brazilians | NR | Clinically significant depressive symptoms | 13 | 14,720 | 26 |
| **Bernard,** **2017 [11]** | PLoS One | 2.8 | Untreated or mixed groups of people living with HIV in sub-Saharan Africa | NR | Depression | 3 | 2,581 | 9 |
| **Buchberger, 2016 [12]** | Psychoneuroendocrinology | 4.8 | Children and adolescents with type 1 diabetes | NR | Depressive symptoms | 6 | 1,320 | 30 |
| **Dowlatshahi, 2014 [13]^b^** | Journal of Investigative Dermatology | 7.2 | Psoriasis patients | Questionnaires | Depressive symptoms | 33 | 8,467 | 28 |
| **Falah-Hassani, 2015 [14]** | Journal of Psychiatric Research | 4.5 | Immigrant women | NR | Depressive symptoms | 18 | 13,749 | 20 |
| **Fellmeth, 2016 [15]** | British Journal of Obstetrics and Gynaecology | 5.1 | Migrant women from low- and middle-income countries | A structured tool | Any depressive disorder | 16 | 3,492 | 31 |
| **Ghaemmohamadi, 2018 [16]** | Journal of Affective Disorders | 3.8^c^ | Cardiovascular patients in Iran | NR | Depression | 12 | 9,292 | 47 |
| **Kim, 2015 [17]** | PLoS One | 3.1 | Asian-American adults in non-clinical settings | Center for Epidemiologic Studies Depression Scale | Depression | 18 | 5,356 | 36 |
| **Krebber, 2014 [3]^a^** | Psycho-Oncology | 4.0 | Adult cancer patients during or after treatment | Hospital Anxiety and Depression Scale - Depression with threshold of 8 | Depression | 75 | 27,384 | 17 |
| **Lai, 2017 [18]^b^** | British Journal of Dermatology | 6.1 | Patients with vitiligo | Self-report questionnaires | Depressive symptoms | 12 | 1,080 | 34 |
| **Lei, 2016 [19]** | PLoS One | 2.8 | Chinese university students | NR | Depression | 39 | 32,694 | 24 |
| **Li, 2014 [20]** | Archives of Gerontology and Geriatrics | 1.9 | Chinese older adults | 30-item Geriatric Depression Scale or the Center for Epidemiologic Studies Depression Scale | Depressive symptoms | 81 | 88,417 | 24 |
| **Li, 2015 [21]^b^** | Medicine | 2.1 | Patients with hypertension | Self-rating scales | Depressive symptoms | 27 | 10,194 | 30 |
| **Luppa, 2012 [22]^b^** | Journal of Affective Disorders | 3.3 | Community-based elderly population aged 75 years and older | NR | Depressive disorders | 12 | 57,710 | 17 |
| **Masoumi, 2013 [23]** | Iranian Journal of Public Health | 0.6 | Infertile couples in Iran | NR | Depression | 12 | 2,818 | 47 |
| **Matcham, 2013 [24]^b^** | Rheumatology | 4.4 | Patients with rheumatoid arthritis | Patient Health Questionnaire-9 | Depression | 2 | 659 | 39 |
| **Muscatelli, 2017 [25]** | Journal of Orthopaedic Trauma | 2.4 | Patients who experienced acute orthopaedic trauma to the appendicular skeleton or pelvis | NR | Depression | 22 | 6,005 | 33 |
| **Osinubi, 2018 [26]^b^** | British Journal of Dermatology | 6.1^c^ | People with vitiligo | Questionnaires | Depression | 11 | 892 | 29 |
| **Pacheco, 2017 [27]** | Revista Brasileira de Psiquiatria | 2.1 | Medical students in Brazil | NR | Depression | 25 | 9,097 | 31 |
| **Rabiee, 2016 [28]** | Critical Care Medicine | 7.1 | ICU survivors | Validated instrument | Depressive symptoms | 15 | 1,767 | 30 |
| **Ravaghi, 2017 [29]** | Iranian Journal of Kidney Diseases | 1.2 | Iranian hemodialysis patients | NR | Depressive symptoms | 27 | 2,822 | 62 |
| **Ren, 2014 [30]** | Chinese Medical Journal | 1.1 | Coronary heart disease patients | NR | Depression | 23 | 5,236 | 51 |
| **Sajjadi, 2013 [31]** | Global Journal of Health Science | NR | Iranian adolescents | Listed names of questionnaires used | Depression | 12 | 12,851 | 44 |
| **Sarokhani, 2013 [32]** | Depression Research and Treatment | NR | Iranian university students | NR | Depression | 35 | 9,443 | 33 |
| **Scott, 2016 [33]** | Aging & Mental Health | 2.7 | Older adults after total joint replacement | NR | Clinically significant levels of depression | 6 | 1,442 | 23 |
| **Silva, 2014 [6]^a^** | Revista Brasileira de Psiquiatria | 1.8 | Brazilian adults | NR | Depressive symptoms | 15 | 442,482 | 14 |
| **Veisani, 2013 [34]** | Depression Research and Treatment | NR | Postpartum Iranian women | NR | Postpartum depression | 41 | 21,907 | 25 |
| **Volkert, 2013 [7]^a^** | Ageing Research Reviews | 7.6 | Older people in Western countries | NR | Dimensional depression | 21 | 51,236 | 19 |
| **Wang, 2017 [35]^b^** | BMJ Open | 2.4 | Outpatients | Screening instruments | Depression or depressive symptoms | 17 | 4,702 | 36 |
| **Watts, 2015 [36]** | BMJ Open | 2.6 | Patients with ovarian cancer | NR | Depression | 11 | 2,157 | 25 |
| **Yang, 2013 [37]** | BMC Cancer | 3.3 | Chinese adults with cancer | NR | Depression | 17 | 1,692 | 55 |
| **Yuen, 2016 [38]** | AIDS CARE | 1.8 | Female sex workers | NR | Probable depression | 4 | 841 | 62 |
| **Zhang, 2012 [39]** | International Journal of Geriatric Psychiatry | 3.0 | Older adults in China (population aged 60 years and above) | NR | Depressive symptoms | 32 | 31,491 | 23 |
| **Zhang, 2017 [8]^a^** | BMC Psychiatry | 2.4 | Adult systemic lupus erythematosus patients | Hospital Anxiety and Depression Scale with threshold of 8 | Depression | 12 | 1,474 | 30 |

**Abbreviations**: NR: Not reported.

^a^ Also included in Additional Table 1a (Meta-analyses based on diagnostic interviews only).

^b^ Also included in Additional Table 1c (Meta-analyses based on a combination of classification methods).

^c^ Impact factor for year of publication was unavailable on Thompson Reuter website, thus impact factor for the previous year is reported.

**Table S1c. Characteristics of Included Meta-analyses based on a Combination of Classification Methods (Validated Diagnostic Interview, Unstructured Diagnostic Interview, Screening Tool or Rating Scale, Other – e.g., Medical Records)**

| **First author, year** | **Journal** | **Impact factor for year of publication** | **Participant group** | **Classification method terminology in abstract** | **Terminology for prevalence value in abstract** | **N primary studies included** | **N participants** | **Pooled prevalence (%)** | **N (%) included studies with validated diagnostic interview** | **N (%) included studies with unstructured diagnostic interview** | **N (%) included studies with screening tool or rating scale** |
| --- | --- | --- | --- | --- | --- | --- | --- | --- | --- | --- | --- |
| **Attanayake, 2009 [40]** | Medicine, Conflict and Survival | NR | Children exposed to war | NR | Depression | 4 | 197 | 43 | 0 (0) | 1 (25) | 3 (75) |
| **Ayerbe, 2013 [41]** | The British Journal of Psychiatry | 7.3 | Patients after stroke | NR | Depression | 43 | 19,468 | 29 | 4 (9) | 5 (12) | 33 (77) |
| **Cameron, 2016 [42]** | Journal of Affective Disorders | 3.4 | Men in either the prenatal or postpartum period (defined as the first 12-months following childbirth) | NR | Depression | 74 | 41,480 | 8 | 5 (7) | 0 (0) | 68 (92) |
| **Chen, 2015 [43]** | Translational Neurodegeneration | NR | Parkinson’s disease patients | NR | Depression | 170 | 35,662 | 37 | 23 (14) | 17 (10) | 108 (64) |
| **Dawes, 2016 [44]** | JAMA | 44.4 | Patients seeking and undergoing bariatric surgery | NR | Depression | 34 | 51,908 | 19 | 11 (32) | 2 (6) | 14 (41) |
| **Douglas, 2014 [45]** | Bipolar Disorders | 5.0 | Pre-pubertal children | NR | Major depression | 11 | 15,477 | 1 | 5 (45) | 1 (9) | 2 (18) |
| **Dowlatshahi, 2014 [13]^a^** | Journal of Investigative Dermatology | 7.2 | Psoriasis patients | International Classification of Diseases codes | Clinical depression | 10 | 201,469 | 12 | 0 (0) | 1 (10) | 0 (0) |
| **Fazel, 2008 [46]** | Journal of the American Academy of Child and Adolescent Psychiatry | 4.8 | Boys in juvenile detention and correctional facilities | NR | Major depression | 16 | 3,323 | 11 | 12 (75) | 2 (13) | 2 (13) |
| **Ferrari, 2013 [47]** | Psychological Medicine | 5.4 | Population-based samples | NR | Major depressive disorder | 82 | 495,229 | 5 | 55 (67) | 4 (5) | 23 (28) |
| **Fiest, 2013 [48]** | Neurology | 8.3 | Persons with epilepsy | NR | Active (current or past year) depression | 9 | 29,891 | 23 | 0 (0) | 0 (0) | 5 (56) |
| **Fu, 2017 [49]** | Oncotarget | 5.2^b^ | Rheumatoid arthritis patients in China | NR | Depression | 21 | 4,447 | 48 | 0 (0) | 0 (0) | 18 (86) |
| **Gu, 2013 [50]** | PLoS One | 3.5 | People in mainland China | NR | Major depressive disorder | 14 | 163,998 | 2 | 0 (0) | 0 (0) | 0 (0) |
| **Lai, 2017 [18]^a^** | British Journal of Dermatology | 6.1 | Patients with vitiligo | Diagnostic codes | Depression | 3 | 236 | 25 | 0 (0) | 1 (33) | 0 (0) |
| **Li, 2015 [21]^a^** | Medicine | 2.1 | Patients with hypertension | Clinical interviews and self-rating scales | Depression | 41 | 30,796 | 27 | 3 (7) | 1 (2) | 36 (88) |
| **Lindert, 2009 [51]** | Social Science & Medicine | 2.7 | Labor migrants | NR | Depression | 9 | 12,019 | 20 | 6 (67) | 0 (0) | 3 (33) |
| **Loh, 2017 [52]** | Journal of the American Medical Directors Association | 5.3 | Caregivers of stroke survivors | NR | Depressive symptoms | 11 | 1,472 | 40 | 0 (0) | 0 (0) | 10 (91) |
| **Luppa, 2012 [22]^a^** | Journal of Affective Disorders | 3.3 | Community-based elderly population aged 75 years and older | NR | Major depression | 3 | 4,804 | 7 | 2 (67) | 0 (0) | 1 (33) |
| **Mata, 2015 [53]** | JAMA | 37.7 | Resident physicians | Clinical interviews and self-report instruments | Depression or depressive symptoms | 54 | 17,560 | 29 | 1 (2) | 2 (4) | 51 (94) |
| **Matcham, 2013 [24]^a^** | Rheumatology | 4.4 | Patients with rheumatoid arthritis | NR | Major depressive disorder | 4 | 480 | 17 | 3 (75) | 0 (0) | 0 (0) |
| **Mitchell, 2011 [54]** | Lancet Oncology | 22.6 | Adults with cancer in palliative-care settings | Psychiatric interviews | Depression defined by the Diagnostic and Statistical Manual of Mental Disorders (DSM) or International Classification of Diseases (ICD) criteria | 23 | 3,372 | 17 | 20 (87) | 1 (4) | 2 (9) |
| **Mitchell, 2013 [55]** | Lancet Oncology | 24.7 | Patients diagnosed with cancer at least 2 years previously | NR | Depression | 16 | 51,381 | 12 | 0 (0) | 2 (13) | 12 (75) |
| **Osinubi, 2018 [26]^a^** | British Journal of Dermatology | 6.1 | People with vitiligo | NR | Clinically diagnosed depression | 9 | 592 | 21 | 1 (11) | 3 (33) | 0 (0) |
| **Özcan, 2017 [56]** | Archives of Psychiatric Nursing | 1.1 | Postpartum women from Turkey | NR | Postpartum depression | 52 | 17,019 | 24 | 1 (2) | 0 (0) | 51 (98) |
| **Palmer, 2013 [57]** | Kidney International | 8.5 | Patients with stage 5D chronic kidney disease | Interview | Depression | 28 | 2,855 | 23 | 17 (61) | 4 (14) | 5 (18) |
| **Palmer, 2013 [58]** | American Journal of Kidney Diseases | 5.8 | Adults with chronic kidney disease | Depression status as determined by physician diagnosis, clinical coding, or self-reported scales | Depression | 22 | 83,381 | 27 | 2 (9) | 0 (0) | 13 (59) |
| **Paulson, 2010 [59]** | JAMA | 30.0 | Fathers between the first trimester of pregnancy and the first postpartum year | NR | Depression | 43 | 28,004 | 10 | 2 (5) | 0 (0) | 41 (95) |
| **Rotenstein, 2016 [60]** | JAMA | 44.4 | Medical students | Validated questionnaire or structured interview | Depression or depressive symptoms | 183 | 122,356 | 27 | 1 (1) | 0 (0) | 182 (99) |
| **Sadeghirad, 2010 [61]** | International Journal of Preventive Medicine | NR | Iranian people | NR | Major depressive disorder | 23 | 24,093 | 4 | 0 (0) | 0 (0) | 0 (0) |
| **Shanmugasegaram, 2012 [62]** | Maturitas | 2.8 | Women with coronary artery disease | Structured clinical interviews | Major depression | 8 | 509 | 19 | 7 (88) | 0 (0) | 1 (13) |
| **Soysal, 2017 [63]** | Ageing Research Reviews | 9.0 | Older adults | NR | Depression | 11 | 8,023 | 39 | 1 (9) | 0 (0) | 10 (91) |
| **Tao, 2018 [64]** | AIDS and Behavior | 3.0^b^ | People living with HIV | NR | Depression | 9 | 7,375 | 41 | 0 (0) | 1 (11) | 7 (78) |
| **Upadhyay, 2017 [65]** | Bulletin of the World Health Organization | 6.4 | Indian mothers | NR | Postpartum depression | 38 | 20,043 | 22 | 2 (5) | 3 (8) | 33 (87) |
| **Wang, 2017 [35]^a^** | BMJ Open | 2.4 | Outpatients | NR | Depression or depressive symptoms | 83 | 41,344 | 27 | 4 (5) | 1 (1) | 73 (88) |
| **Williams, 2015 [66]** | Archives of Physical Medicine and Rehabilitation | 3.0 | Persons with spinal cord injury | Diagnostic measure of depression (i.e., an unstructured, semi-structured, or structured clinical interview, and/or a clinician diagnosis) | Depression | 21 | 35,893 | 22 | 7 (33) | 4 (19) | 3 (14) |
| **Younossi, 2016 [67]** | Gastroenterology | 18.4 | Hepatitis C patients | NR | Depression | 12 | 130,039 | 25 | 4 (33) | 1 (8) | 4 (33) |
| **Zhang, 2011 [68]** | General Hospital Psychiatry | 2.7 | Chronic obstructive pulmonary disease patients | NR | Depressive symptoms | 8 | 39,587 | 25 | 1 (13) | 0 (0) | 6 (75) |
| **Zheng, 2017 [69]** | Scientific Reports | 4.1 | Eye disease patients | NR | Depression or depressive symptoms | 28 | 6,589 | 25 | 1 (4) | 0 (0) | 25 (89) |

**Abbreviations**: NR: Not reported.

^a^ Also included in Additional Table 1b (Meta-analyses based on depression screening tools and rating scales only).

^b^ Impact factor for year of publication was unavailable on Thompson Reuter website, thus impact factor for the previous year is reported.

**Table S1d.** **Characteristics of Included Meta-analyses Published in Journals with Impact Factor ≥ 10 (All were based on a Combination of Classification Methods (Validated Diagnostic Interview, Unstructured Diagnostic Interview, Screening Tool or Rating Scale, Other – e.g., Medical Records))**

| **First author, year** | **Journal** | **Impact factor for year of publication** | **Participant group** | **Classification method terminology in abstract** | **Terminology for prevalence value in abstract** | **N primary studies included** | **N participants** | **Pooled prevalence (%)** | **N (%) included studies with validated diagnostic interview** | **N (%) included studies with unstructured diagnostic interview** | **N (%) included studies with screening tool or rating scale** |
| --- | --- | --- | --- | --- | --- | --- | --- | --- | --- | --- | --- |
| **Dawes, 2016 [13]** | JAMA | 44.4 | Patients seeking and undergoing bariatric surgery | NR | Depression | 34 | 51,908 | 19 | 11 (32) | 2 (6) | 14 (41) |
| **Mata, 2015 [15]** | JAMA | 37.7 | Resident physicians | Clinical interviews and self-report instruments | Depression or depressive symptoms | 54 | 17,560 | 29 | 1 (2) | 2 (4) | 51 (94) |
| **Mitchell, 2011 [84]** | Lancet Oncology | 22.6 | Adults with cancer in palliative-care settings | Psychiatric interviews | Depression defined by the Diagnostic and Statistical Manual of Mental Disorders or International Classification of Diseases criteria | 23 | 3,372 | 17 | 20 (87) | 1 (4) | 2 (9) |
| **Mitchell, 2013 [85]** | Lancet Oncology | 24.7 | Patients diagnosed with cancer at least 2 years previously | NR | Depression | 16 | 51,381 | 12 | 0 (0) | 2 (13) | 12 (75) |
| **Paulson, 2010 [14]** | JAMA | 30.0 | Fathers between the first trimester of pregnancy and the first postpartum year | NR | Depression | 43 | 28,004 | 10 | 2 (5) | 0 (0) | 41 (95) |
| **Rotenstein, 2016 [16]** | JAMA | 44.4 | Medical students | Validated questionnaire or structured interview | Depression or depressive symptoms | 183 | 122,356 | 27 | 1 (1) | 0 (0) | 182 (99) |
| **Younossi, 2016 [95]** | Gastroenterology | 18.4 | Hepatitis C patients | NR | Depression | 12 | 130,039 | 25 | 4 (33) | 1 (8) | 4 (33) |

**Figure S1a:** Forest Plot of Pooled Prevalence Estimates from Meta-analyses based on Diagnostic Interviews Only


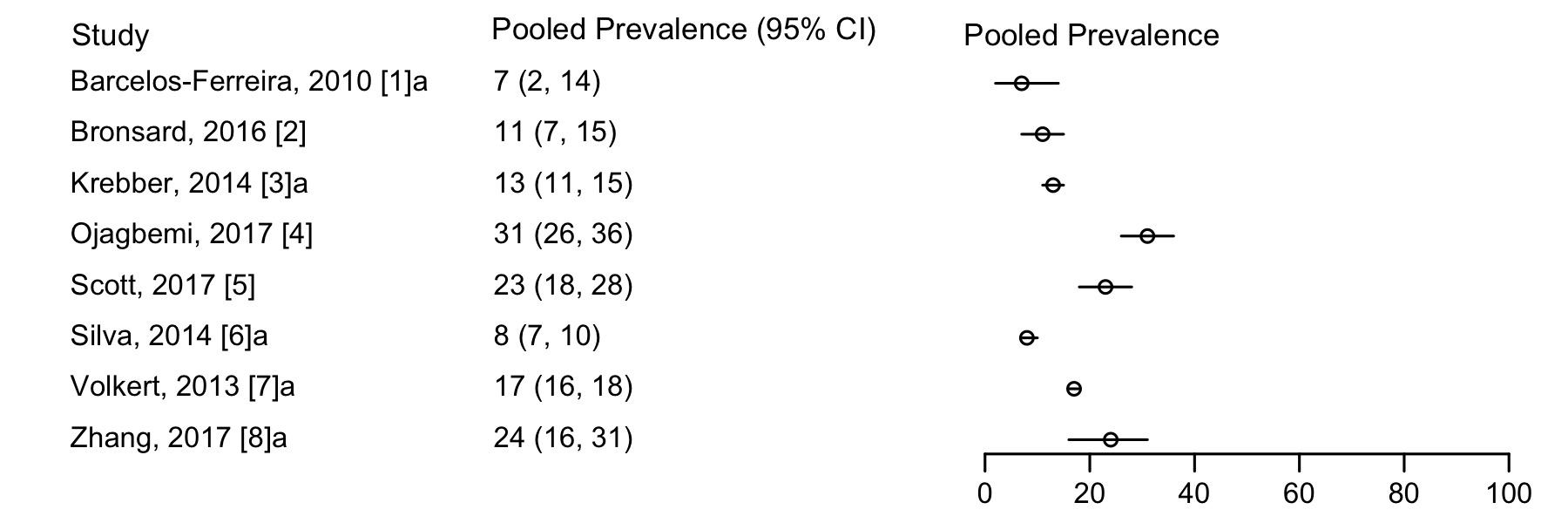


**Abbreviations**: CI: Confidence interval.

^a^ Also included in Additional Figure 1b (Meta-analyses based on depression screening tools and rating scales only).

**Figure S1b:** Forest Plot of Pooled Prevalence Estimates from Meta-analyses based on Screening Tools and Rating Scales Only


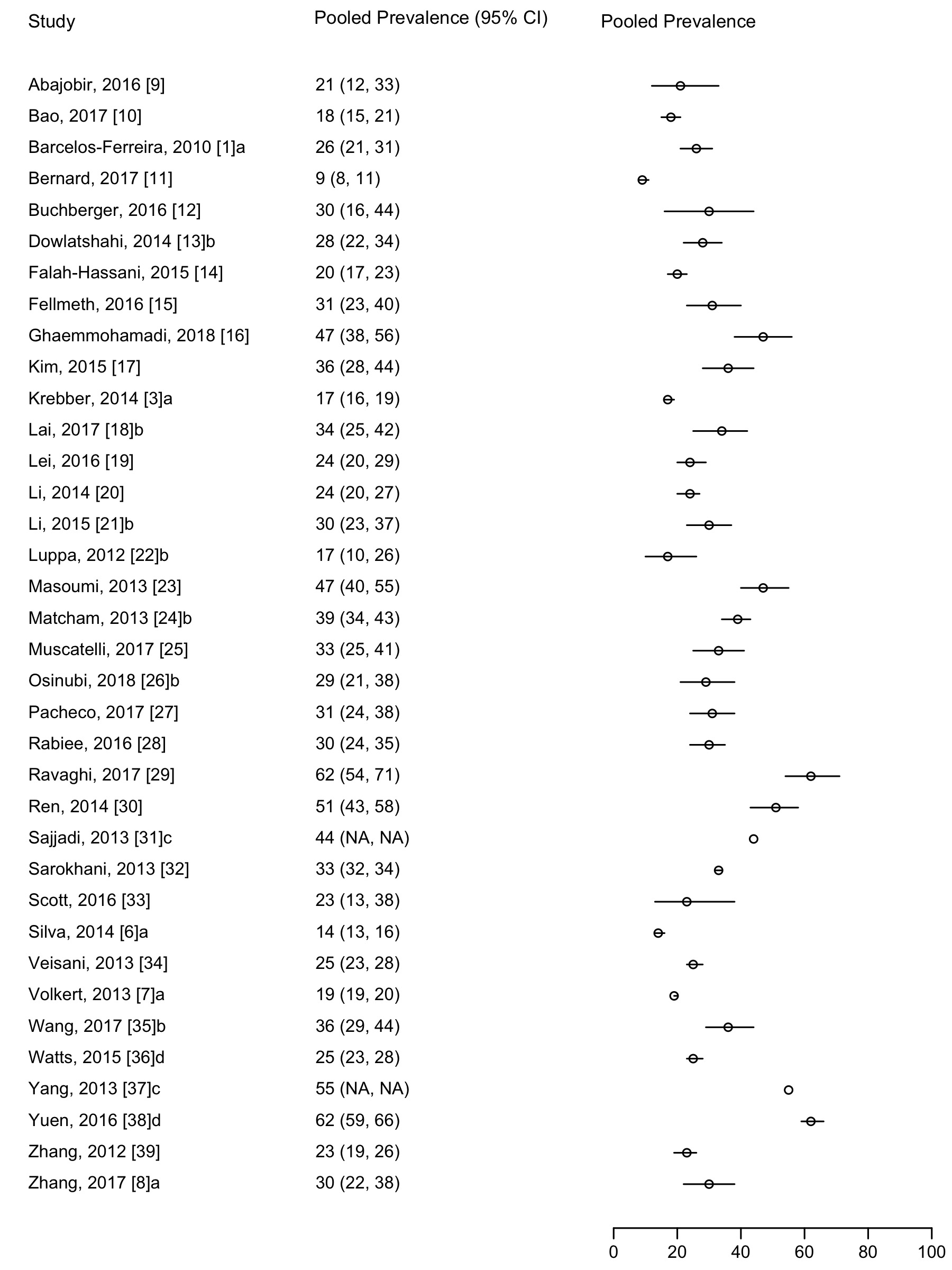


**Abbreviations**: CI: Confidence interval, NA: Not available.

^a^ Also included in Additional Figure 1a (Meta-analyses based on diagnostic interviews only).

^b^ Also included in Additional Figure 1c (Meta-analyses based on a combination of classification methods).

^c^ Article did not report confidence interval for pooled prevalence value.

^d^ Confidence interval is from fixed-effects meta-analysis (all others are from random-effects meta-analysis).

**Figure S1c:** Forest Plot of Pooled Prevalence Estimates from Meta-analyses based on a Combination of Classification Methods (Validated Diagnostic Interview, Unstructured Diagnostic Interview, Screening Tool or Rating Scale, Other – e.g., Medical Records)


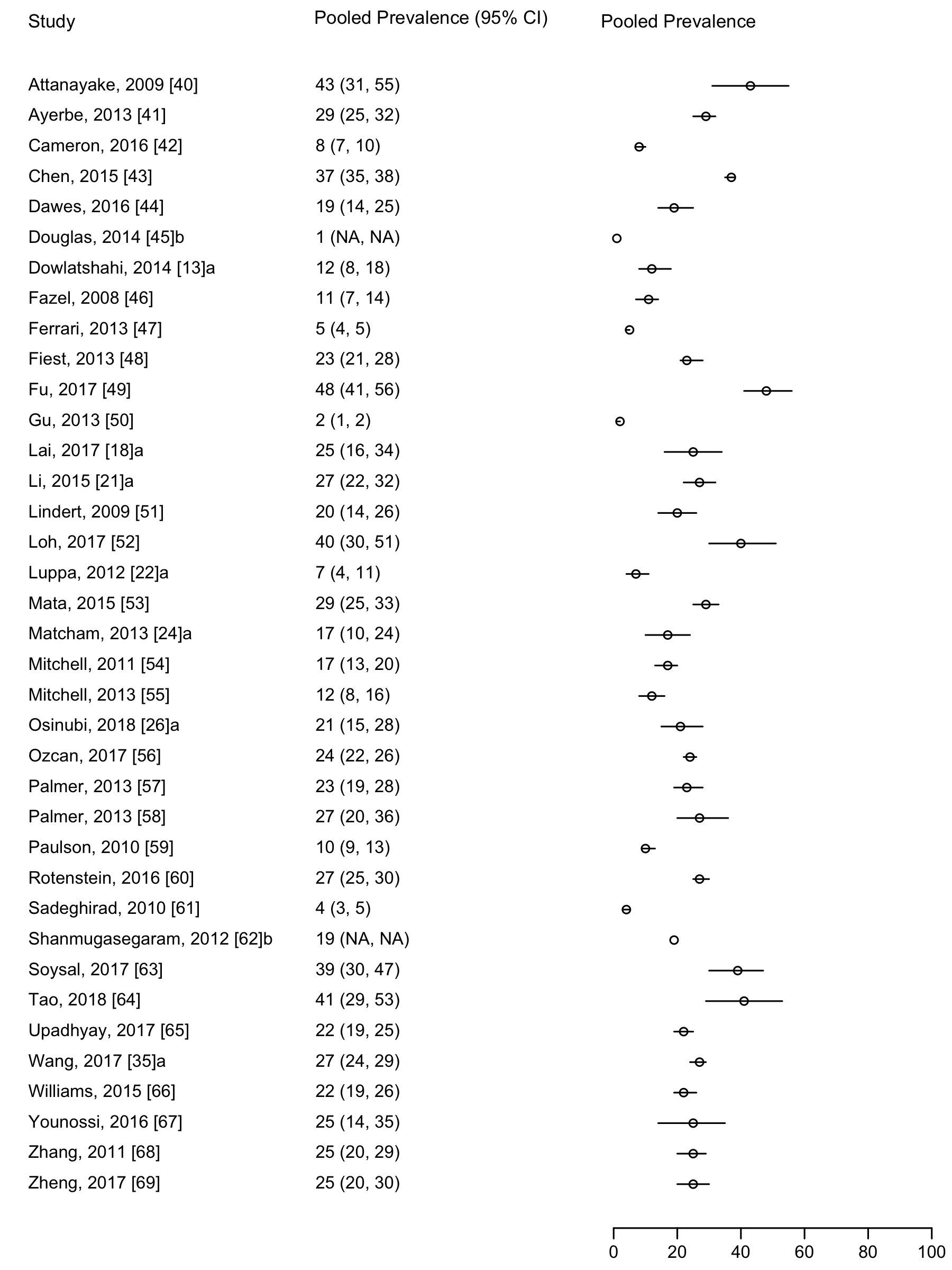


**Abbreviations**: CI: Confidence interval, NA: Not available.

^a^ Also included in Additional Figure 1a (Meta-analyses based on diagnostic interviews only).

^b^ Article did not report confidence interval for pooled prevalence value.

**Figure S2a:** Forest Plots of Pooled Prevalence Values from Studies with Meta-analyses based on Screening Tools and Rating Scales Only and Meta-analyses based on Diagnostic Interviews Only


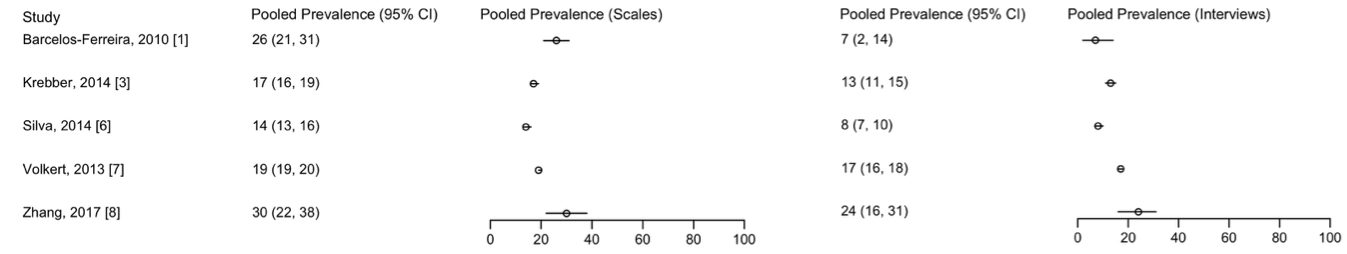


**Figure S2b:** Forest Plots of Pooled Prevalence Values from Studies with Meta-analyses based on Screening Tools and Rating Scales Only and Meta-analyses based on a Combination of Classification Methods (Validated Diagnostic Interview, Unstructured Diagnostic Interview, Screening Tool or Rating Scale, Other – e.g., Medical Records)

**
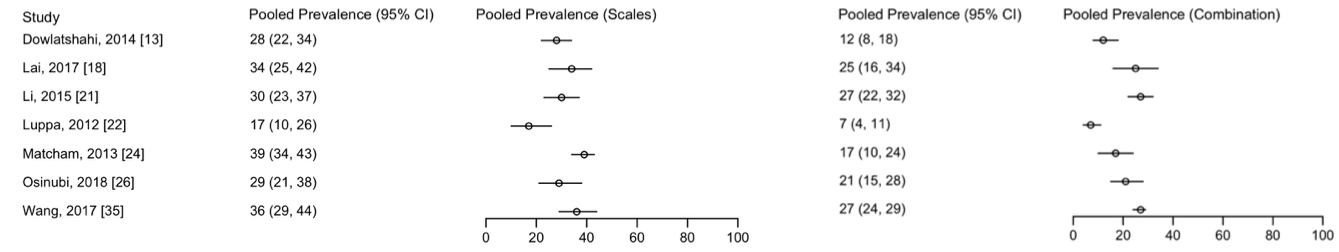
**

**References FOR INCLUDED PRIMARY STUDIES**

1. Barcelos-Ferreira R, Izbicki R, Steffens DC, Bottino CM. Depressive morbidity and gender in community-dwelling Brazilian elderly: systematic review and meta-analysis. *Int Psychogeriatr* 2010;22:712-726.
2. Bronsard G, Alessandrini M, Fond G, et al. The prevalence of mental disorders among children and adolescents in the child welfare system: a systematic review and meta-analysis. *Medicine* 2016;95:e2622.
3. Krebber AM, Buffart LM, Kleijn G, et al. Prevalence of depression in cancer patients: a meta-analysis of diagnostic interviews and self-report instruments. *Psychooncology* 2014;23:121-130.
4. Ojagbemi A, Akpa O, Elugbadebo F, Owolabi M, Ovbiagele B. Depression after stroke in Sub-Saharan Africa: a systematic review and meta-analysis. *Behav Neurol* 2017;2017:4160259.
5. Scott AJ, Sharpe L, Hunt C, Gandy M. Anxiety and depressive disorders in people with epilepsy: a meta-analysis. *Epilepsia* 2017;58:973-982.
6. Silva MT, Galvao TF, Martins SS, Pereira MG. Prevalence of depression morbidity among Brazilian adults: a systematic review and meta-analysis. *Rev Bras Psiquiatr* 2014;36:262-270.
7. Volkert J, Schulz H, Härter M, Wlodarczyk O, Andreas S. The prevalence of mental disorders in older people in Western countries - a meta-analysis. *Ageing Res Rev* 2013;12:339-53.
8. Zhang L, Fu T, Yin R, Zhang Q, Shen B. Prevalence of depression and anxiety in systemic lupus erythematosus: a systematic review and meta-analysis. *BMC Psychiatry* 2017;17:70.
9. Abajobir AA, Maravilla JC, Alati R, Najman JM. A systematic review and meta-analysis of the association between unintended pregnancy and perinatal depression. *J Affect Disord* 2016;192:56-63.
10. Bao YP, Han Y, Ma J, et al. Cooccurrence and bidirectional prediction of sleep disturbances and depression in older adults: meta-analysis and systematic review. *Neurosci Biobehav Rev* 2017;75:257-273.
11. Bernard C, Dabis F, de Rekeneire N. Prevalence and factors associated with depression in people living with HIV in sub-Saharan Africa: a systematic review and meta-analysis. *PloS One* 2017;12:e0181960.
12. Buchberger B, Huppertz H, Krabbe L, Lux B, Mattivi JT, Siafarikas A. Symptoms of depression and anxiety in youth with type 1 diabetes: A systematic review and meta-analysis. *Psychoneuroendocrinology* 2016;70:70-84.
13. Dowlatshahi EA, Wakkee M, Arends LR, Nijsten T. The prevalence and odds of depressive symptoms and clinical depression in psoriasis patients: a systematic review and meta-analysis. *J Invest Dermatol* 2014;134:1542-1551.
14. Falah-Hassani K, Shiri R, Vigod S, Dennis CL. Prevalence of postpartum depression among immigrant women: a systematic review and meta-analysis. *J Psychiatr Res* 2015;70:67-82.
15. Fellmeth G, Fazel M, Plugge E. Migration and perinatal mental health in women from low- and middle-income countries: a systematic review and meta-analysis. *BJOG* 2017;124:742-752.
16. Ghaemmohamadi MS, Behzadifar M, Ghashghaee A, et al. Prevalence of depression in cardiovascular patients in Iran: a systematic review and meta-analysis from 2000 to 2017. *J Affect Disord* 2018;227:149-155.
17. Kim HJ, Park E, Storr CL, Tran K, Juon HS. Depression among Asian-American adults in the community: systematic review and meta-analysis. *PLoS One* 2015;10:e0127760.
18. Lai YC, Yew YW, Kennedy C, Schwartz RA. Vitiligo and depression: a systematic review and meta-analysis of observational studies. *Br J Dermatol* 2017;177:708-718.
19. Lei XY, Xiao LM, Liu YN, Li YM. Prevalence of depression among Chinese university students: a meta-analysis. *PLoS One* 2016;11:e0153454.
20. Li D, Zhang DJ, Shao JJ, Qi XD, Tian L. A meta-analysis of the prevalence of depressive symptoms in Chinese older adults. *Arch Gerontol Geriatr* 2014;58:1-9.
21. Li Z, Li Y, Chen L, Chen P, Hu Y. Prevalence of depression in patients with hypertension: a systematic review and meta-analysis. *Medicine* 2015;94:e1317.
22. Luppa M, Sikorski C, Luck T, et al. Age- and gender-specific prevalence of depression in latest-life--systematic review and meta-analysis. *J Affect Disord* 2012;136:212-221.
23. Masoumi SZ, Poorolajal J, Keramat A, Moosavi SA. Prevalence of depression among infertile couples in Iran: a meta-analysis study. *Iran J Public Health* 2013;42:458-466.
24. Matcham F, Rayner L, Steer S, Hotopf M. The prevalence of depression in rheumatoid arthritis: a systematic review and meta-analysis. *Rheumatology* 2013;52:2136-2148.
25. Muscatelli S, Spurr H, OʼHara NN, OʼHara LM, Sprague SA, Slobogean GP. Prevalence of depression and posttraumatic stress disorder after acute orthopaedic trauma: a systematic review and meta-analysis. *J Orthop Trauma* 2017;31:47-55.
26. Osinubi O, Grainge MJ, Hong L, et al. The prevalence of psychological comorbidity in people with vitiligo: a systematic review and meta-analysis. *Br J Dermatol* 2018;178:863-878.
27. Pacheco JP, Giacomin HT, Tam WW, et al. Mental health problems among medical students in Brazil: a systematic review and meta-analysis. *Rev Bras Psiquiatr* 2017;39:369-378.
28. Rabiee A, Nikayin S, Hashem MD, et al. Depressive symptoms after critical illness: a systematic review and meta-analysis. *Crit Care Med* 2016;44:1744-1753.
29. Ravaghi H, Behzadifar M, Behzadifar M, et al. Prevalence of depression in hemodialysis patients in Iran: a systematic review and meta-analysis. *Iran J Kidney Dis* 2017;11:90-98.
30. Ren Y, Yang H, Browning C, Thomas S, Liu M. Prevalence of depression in coronary heart disease in China: a systematic review and meta-analysis. *Chin Med J* 2014;127:2991-2998.
31. Sajjadi H, Kamal SH, Rafiey H et al. A systematic review of the prevalence and risk factors of depression among Iranian adolescents. *Glob J Health Sci* 2013;5:16-27.
32. Sarokhani D, Delpisheh A, Veisani Y, Sarokhani MT, Manesh RE, Sayehmiri K. Prevalence of depression among university students: a systematic review and meta-analysis Study. *Depress Res Treat* 2013;2013:373857.
33. Scott JE, Mathias JL, Kneebone AC. Depression and anxiety after total joint replacement among older adults: a meta-analysis. *Aging Ment Health* 2016;20:1243-1254.
34. Veisani Y, Delpisheh A, Sayehmiri K, Rezaeian S. Trends of postpartum depression in Iran: a systematic review and meta-analysis. *Depress Res Treat* 2013;2013:291029.
35. Wang J, Wu X, Lai W, et al. Prevalence of depression and depressive symptoms among outpatients: a systematic review and meta-analysis. *BMJ Open* 2017;7:e017173.
36. Watts S, Prescott P, Mason J, McLeod N, Lewith G. Depression and anxiety in ovarian cancer: a systematic review and meta-analysis of prevalence rates. *BMJ Open* 2015;5:e007618.
37. Yang YL, Liu L, Wang Y, et al. The prevalence of depression and anxiety among Chinese adults with cancer: a systematic review and meta-analysis. *BMC Cancer* 2013;13:393.
38. Yuen WW, Tran L, Wong CK, Holroyd E, Tang CS, Wong WC. Psychological health and HIV transmission among female sex workers: a systematic review and meta-analysis. *AIDS Care* 2016;28:816-824.
39. Zhang L, Xu Y, Nie H, Zhang Y, Wu Y. The prevalence of depressive symptoms among the older in China: a meta-analysis. *Int J Geriatr Psychiatry* 2012;27:900-906.
40. Attanayake V, McKay R, Joffres M, Singh S, Burkle F Jr, Mills E. Prevalence of mental disorders among children exposed to war: a systematic review of 7,920 children. *Med Confl Surviv* 2009;25:4-19.
41. Ayerbe L, Ayis S, Wolfe CD, Rudd AG. Natural history, predictors and outcomes of depression after stroke: systematic review and meta-analysis. *Br J Psychiatry* 2013;202:14-21.
42. Cameron EE, Sedov ID, Tomfohr-Madsen LM. Prevalence of paternal depression in pregnancy and the postpartum: an updated meta-analysis. *J Affect Disord* 2016;206:189-203.
43. Chen H, Zhao EJ, Zhang W, et al. Meta-analyses on prevalence of selected Parkinson's nonmotor symptoms before and after diagnosis. *Transl Neurodegener* 2015;4:1.
44. Dawes AJ, Maggard-Gibbons M, et al. Mental health conditions among patients seeking and undergoing bariatric surgery: a meta-analysis. *JAMA* 2016;315:150-163.
45. Douglas J, Scott J. A systematic review of gender-specific rates of unipolar and bipolar disorders in community studies of pre-pubertal children. *Bipolar Disord* 2014;16:5-15.
46. Fazel S, Doll H, Långström N. Mental disorders among adolescents in juvenile detention and correctional facilities: a systematic review and metaregression analysis of 25 surveys. *J Am Acad Child Adolesc Psychiatry* 2008;47:1010-1019.
47. Ferrari AJ, Somerville AJ, Baxter AJ, et al. Global variation in the prevalence and incidence of major depressive disorder: a systematic review of the epidemiological literature. *Psychol Med* 2013;43:471-481.
48. Fiest KM, Dykeman J, Patten SB, et al. Depression in epilepsy: a systematic review and meta-analysis. *Neurology* 2013;80:590-599.
49. Fu X, Li ZJ, Yang CJ, et al. prevalence of depression in rheumatoid arthritis in China: a systematic review. *Oncotarget* 2017;8:53623-53630.
50. Gu L, Xie J, Long J, et al. Epidemiology of major depressive disorder in mainland China: a systematic review. *PloS One* 2013;8:e65356.
51. Lindert J, Ehrenstein OS, Priebe S, Mielck A, Brähler E. Depression and anxiety in labor migrants and refugees--a systematic review and meta-analysis. *Soc Sci Med* 2009;69:246-257.
52. Loh AZ, Tan JS, Zhang MW, Ho RC. The global prevalence of anxiety and depressive symptoms among caregivers of stroke survivors. *J Am Med Dir Assoc* 2017;18:111-116.
53. Mata DA, Ramos MA, Bansal N, et al. Prevalence of depression and depressive symptoms among resident physicians: a systematic review and meta-analysis. *JAMA* 2015;314:2373-2383.
54. Mitchell AJ, Chan M, Bhatti H, et al. Prevalence of depression, anxiety, and adjustment disorder in oncological, haematological, and palliative-care settings: a meta-analysis of 94 interview-based studies. *Lancet Oncol* 2011;12:160-174.
55. Mitchell AJ, Ferguson DW, Gill J, Paul J, Symonds P. Depression and anxiety in long-term cancer survivors compared with spouses and healthy controls: a systematic review and meta-analysis. *Lancet Oncol* 2013;14:721-32.
56. Özcan NK, Boyacıoğlu NE, Dinç H. Postpartum depression prevalence and risk factors in Turkey: a systematic review and meta-analysis. *Arch Psychiatr Nurs* 2017;31:420-428.
57. Palmer S, Vecchio M, Craig JC, et al. Prevalence of depression in chronic kidney disease: systematic review and meta-analysis of observational studies. *Kidney Int* 2013;84:179-191.
58. Palmer SC, Vecchio M, Craig JC, et al. Association between depression and death in people with CKD: a meta-analysis of cohort studies. *Am J Kidney Dis* 2013;62:493-505.
59. Paulson JF, Bazemore SD. Prenatal and postpartum depression in fathers and its association with maternal depression: a meta-analysis. *JAMA* 2010;303:1961-1969.
60. Rotenstein LS, Ramos MA, Torre M, et al. Prevalence of depression, depressive symptoms, and suicidal ideation among medical students: a systematic review and meta-analysis. *JAMA* 2016;316:2214-2236.
61. Sadeghirad B, Haghdoost AA, Amin-Esmaeili M, et al. Epidemiology of major depressive disorder in Iran: a systematic review and meta-analysis. *Int J Prev Med* 2010;1:81-91.
62. Shanmugasegaram S, Russell KL, Kovacs AH, Stewart DE, Grace SL. Gender and sex differences in prevalence of major depression in coronary artery disease patients: a meta-analysis. *Maturitas* 2012;73:305-311.
63. Soysal P, Veronese N, Thompson T et al. Relationship between depression and frailty in older adults: A systematic review and meta-analysis. *Ageing Res Rev* 2017;36:78-87.
64. Tao J, Vermund SH, Qian HZ. Association between depression and antiretroviral therapy use among people living with HIV: a meta-analysis. *AIDS Behav* 2018;22:1542-1550.
65. Upadhyay RP, Chowdhury R, Salehi A, et al. Postpartum depression in India: a systematic review and meta-analysis. *Bull World Health Organ* 2017;95:706-717C.
66. Williams R, Murray A. Prevalence of depression after spinal cord injury: a meta-analysis. *Arch Phys Med Rehabil* 2015;96:133-140.
67. Younossi Z, Park H, Henry L, Adeyemi A, Stepanova M. Extrahepatic manifestations of Hepatitis C: a meta-analysis of prevalence, quality of life, and economic burden. *Gastroenterology* 2016;150:1599-1608.
68. Zhang MW, Ho RC, Cheung MW, Fu E, Mak A. Prevalence of depressive symptoms in patients with chronic obstructive pulmonary disease: a systematic review, meta-analysis and meta-regression. *Gen Hosp Psychiatry* 2011;33:217-223.
69. Zheng Y, Wu X, Lin X, Lin H. The prevalence of depression and depressive Symptoms among eye disease patients: a systematic review and meta-analysis. *Sci Rep* 2017;7:46453.
